# High prevalence of cardiovascular risk factors in adults living in Greece: the EMENO National Health Examination Survey

**DOI:** 10.1186/s12889-020-09757-4

**Published:** 2020-11-07

**Authors:** Giota Touloumi, Argiro Karakosta, Natasa Kalpourtzi, Magda Gavana, Apostolos Vantarakis, Maria Kantzanou, Christos Hajichristodoulou, Grigoris Chlouverakis, Grigoris Trypsianis, Paraskevi V. Voulgari, Yannis Alamanos, Konstantinos Makrilakis, Stavros Liatis, Stylianos Chatzipanagiotou, George Stergiou, on behalf of the EMENO study group, Alamanos Yannis, Benos Alexis, Chlouverakis Grigoris, Hajichristodoulou Christos, Karakatsani Anna, Stergiou George, Touloumi Giota, Trypsianis Grigoris, Vantarakis Apostolos, Voulgari Paraskevi, Karakosta Argiro, Pantazis Nikos, Vourli Georgia, Kalpourtzi Natasa, Katsouyanni Klea, Kantzanou Maria, Chrysochoou Xenia, Gavana Magda, Haidich Bettina, Hadjichristodoulou Christos, Rachiotis George, Voulgari V. Paraskevi, Nikolakopoulos Ilias, Panagiotis Koustenis, Makrilakis Konstantinos, Liatis Stavros

**Affiliations:** 1 Department of Hygiene, Epidemiology, & Medical Statistics, Medical School, National and Kapodistrian University of Athens, 75 Mikras Asias Street, 11527 Athens, Greece; 2 Department of Primary Health Care, General Practice and Health Services Research, Medical School of Aristotle University, Thessaloniki, Greece; 3 Public Health, Medical School, University of Patras, Patra, Greece; 4 Department of Hygiene and Epidemiology, Medical Faculty, University of Thessaly, Larisa, Greece; 5 Laboratory of Biostatistics, School of Medicine, University of Crete, Crete, Greece; 6 Laboratory of Medical Statistics, Medical School, Democritus University of Thrace, Thrace, Greece; 7 Department of Internal Medicine, Medical School, University of Ioannina, Ioannina, Greece; 8 Institute of Epidemiology, Preventive Medicine and Public Health, Corfu, Greece; 9 1st Dept of Propaedeutic Internal Medicine, Medical School, National and Kapodistrian University of Athens, Athens, Greece; 10 Hellenic Diabetes Association (HDA), Athens, Greece; 11 Department of Medical Biopathology, Medical School, National and Kapodistrian University of Athens, Eginition Hospital, Athens, Greece; 12 Hypertension Center STRIDE-7, 3rd Department of Medicine, Medical School, National and Kapodistrian University of Athens, Sotiria Hospital, Athens, Greece

**Keywords:** Cardiovascular disease, Hypertension, Diabetes, Obesity, Hypercholesterolemia, Health examination survey, Population-based study

## Abstract

**Background:**

Nationwide data on cardiovascular risk factors prevalence is lacking in Greece. This work presents the findings of the national health examination survey EMENO (2013–2016) regarding the prevalence of hypertension, hypercholesterolemia, diabetes, obesity and smoking.

**Methods:**

A random sample of adults (≥18 years) was drawn by multistage stratified random sampling based on 2011 Census. All EMENO participants with ≥1 measurement of interest [blood pressure (BP), fasting glucose, HbA1c, total cholesterol (TC), Body Mass Index (BMI)] were included. Hypertension was defined as BP ≥ 140/90 mmHg and/or antihypertensive treatment; diabetes as fasting glucose≥126 mg/dL and/or HbA1c ≥ 6.5% or self-reported diabetes; hypercholesterolemia as TC ≥ 190 mg/dL. Sampling weights were applied to adjust for study design and post-stratification weights to match sample age and sex distribution to population one. Non-response was adjusted by inverse probability weighting.

**Results:**

Of 6006 EMENO participants, 4822 were included (51.5% females, median age:47.9 years). The prevalence of hypertension was 39.2%, higher in men (42.4%) than in women (36.1%); of hypercholesterolemia 60.2%, similar in men (59.5%) and women (60.9%); of diabetes 11.6%, similar men (12.4%) and women (10.9%); of obesity 32.1%, higher in women (33.5% vs 30.2%), although in subjects aged 18–40 year it was higher in men; of current smoking 38.2%, higher in men (44.0%) than in women (32.7%). The prevalence of all risk factors increased substantially with age, except smoking, which followed an inverse U shape.

**Conclusions:**

The burden of cardiovascular risk factors among Greek adults is alarming. There is considerable preventive potential and actions at health care and societal level are urgently needed.

## Background

Cardiovascular diseases (CVD) are the leading cause of mortality worldwide. In 2015, about 17.7 million people died from CVD, representing 31% of all deaths globally [1], producing a total cost greater than $316.6 billion [2]. It is predicted that by 2030 the annual number of deaths attributed to CVD will increase to almost 23.6 million people [3]. In Europe, CVD are responsible for over 3.9 million deaths a year, accounting for 43% of all deaths occurring in Europe, and for 23% of the total disease burden [4].

Common and modifiable risk factors such as hypertension, hypercholesterolemia, diabetes, high body mass index (BMI), smoking, excess alcohol use, unhealthy diet, low physical activity, and their interaction account for about 60% of the CVD deaths [5]. Over the past 30 years age-standardized mortality rates have fallen in most countries albeit to different degrees, with the most striking decreases observed in the USA, Japan and Northern European countries (decline rate 50–60%) [5]. These declines were at least partly related to national preventive programs aiming to control modifiable CVD risk factors. However, in the same period, in Eastern European countries and in Greece, initial increases followed by relatively small reduction in the incidence of CVD were observed [1, 4]. It is assumed that changes towards the modern lifestyle resulted in increases in the prevalence of several CVD risk factors and contributed to these initial increases. Despite reductions in age-standardized mortality, absolute numbers of CVD are increasing due to the aging of the population [4]. This rapid increase imposes large human, social and economic costs, which put additional pressure on the health and social care systems, especially under the present austerity climate across Europe [6].

According to 2014 Hellenic Statistical Authority data, 40% of all deaths in Greece were attributed to CVD. The financial crisis and the subsequently implemented austerity policies in Greece since 2009, have various negative consequences on the populations’ daily life and health, including increase in myocardial infarctions, which have been associated with increased unemployment rates [7].

Until recently, although several notable health surveys had been carried out in Greece, they were restricted to specific regions or high-risk groups [8, 9], or were conducted within specific European projects [10–12]. However, valid, nationwide estimates of the prevalence of chronic diseases including CVD and of associated risk factors are necessary to design and implement prevention strategies. The National Survey of Morbidity and Risk Factors (EMENO) is a nationwide health examination survey focused on cardiovascular and respiratory diseases and their risk factors, performed in a representative sample of the adults living in Greece [13]. In this analysis of the EMENO dataset, we estimated the prevalence of cardiovascular risk factors such as hypertension, hypercholesterolemia, diabetes, increased BMI and smoking in the Greek adult population.

## Methods

### The EMENO survey

Data were derived from the EMENO health examination survey. EMENO is a population-based, cross-sectional survey conducted during May 2013–June 2016. The study design has been described in detail elsewhere [13]. Briefly, a random sample of the adults (≥18 years) living in Greece, excluding institutionalized individuals, homeless and migrants living in detention camps, was drawn by multistage stratified random sampling based on the 2011 Census. Sampling fractions varied by region, with larger fractions in less populated regions. During home visits, trained interviewers administered a standardized questionnaire (available in Greek and English language to facilitate communication with non-Greek speaking participants) to study participants and trained physicians performed physical examinations, collected blood samples and made anthropometric measurements using standardized procedures and equipment [13]. All participants provided signed informed consent. In the rare case that participants were unable to provide a signed informed consent, their legal representatives were asked instead. The study protocol was approved by the Athens University IRB (http://en.uoa.gr/) and the Hellenic Data Protection Authority (www.dpa.gr).

### Measurements

Height and weight were measured without shoes and in light clothes. Arterial blood pressure was measured in sitting position and after at least 5 minutes of rest [13] using an automated (oscillometric) upper-arm cuff device (Microlife BPA 100 Plus), previously successfully validated in adults [14], with appropriate cuff size according to the individual participant’s arm circumference. Three valid consecutive blood pressure measurements were taken with one-minute intervals and the average of the last two was used in the analysis. Smoking, caffeine ingestion during the previous hour, physical exercise during the previous 2 hours and antihypertensive treatment were recorded. All participants were asked to display all drugs there were currently taken to the interviewer, and all drugs were recorded.

Participants were asked to abstain from food and alcohol for at least 8 h, and the patient reported abstinence hours were recorded. Total serum cholesterol, low-density and high-density lipoprotein cholesterol as well as fasting serum glucose and glycated hemoglobin (HbA1c) were determined. For glucose, serum samples were collected in sodium fluoride tubes. Blood samples were centrifuged as soon as possible after venipuncture. After centrifugation, serum samples were stored at -80 °C until shipping to the central laboratory in Athens for further processing. TC in serum, fasting blood glucose in plasma and HbA1c in whole blood were measured by means of ARCHITECT c8000 Clinical Chemistry Analyser (ABBOTT, ABBOTT Park, Illinois, USA). For TC and glucose determination conventional enzymatic photometric assays were used, while HbA1c determination was based on turbidimetric method with an Alkaline Phosphatase conjugated anti-HbA1 antibody.

### Eligibility criteria

All EMENO participants who had at least one measurement of interest [height and weight, systolic (SBP) and diastolic blood pressure (DBP), total serum cholesterol, glucose, HbA1c] were included in the current analysis. Glucose measurements from blood samples taken in individuals who had not been fasted for at least 8 h were excluded.

### Definitions

Hypertension was defined as SBP ≥140 mmHg and/or DBP ≥90 mmHg and/or use of antihypertensive drugs. As various classes of BP medications may be prescribed for other than blood pressure (BP) reasons, in a sensitivity analysis, participants classified as hypertensive based on taking antihypertensive drugs, when not considered as hypertensive if they had not reported a medical history of hypertension. Diabetes was defined as fasting blood glucose ≥126 mg/dL and/or HbA1c ≥6.5% or use of antidiabetic treatment or self-reported diabetes. As part of sensitivity analysis, diabetes prevalence was also estimated using only the fasting glucose criterion or using only the HbA1c criterion. BMI was estimated from measured height and weight. Obesity was defined as BMI ≥30 kg/m^2^, while BMI between 25 kg/m^2^ and 30 Kg/m^2^ was classified as overweight. Hypercholesterolemia was defined as TC levels ≥190 mg/dL and/or use of lipid-lowering agents. TC levels ≥200 or ≥ 240 mg/dL and/or use of lipid-lowering agents were also considered. Smoking status was self-reported and was classified as current-, ex- and never-smoking.

### Statistical analysis

Sampling weights, being the reciprocal of the selection probabilities, were applied to adjust for the sampling design; sampling weights were then multiplied with post stratification weights to match the age, gender and geographical distribution of the sample to that of the Greek population based on the 2011 census provided by the Hellenic Statistical Authority. To adjust for non-response, as a sub-sample of the interviewed individuals participated in the physical examination and provided blood samples, the inverse probability weighting method was applied. Weights were the reciprocal of the response probabilities, estimated through weighted multivariable logistic regression. Weighted means and standard deviations for continuous variables and weighted percentages for categorical variables were provided. Analysis was performed using the svy package in STATA (version 13.0; Stata Corp, College Station, TX).

### Sensitivity analysis

Missing values were imputed using multiple imputations (MI) by the chained equation method. Five imputed datasets were created with 600 burn-in iterations using the mi package in STATA (version 13.0; Stata Corp, College Station, TX). All statistical analyses were performed using the statistical software STATA.

## Results

### Socio-demographic characteristics of the population

In total, 6006 individuals were enrolled in the EMENO study, with the overall response rate being 72.0%. Thirteen individuals with unknown age (necessary for post-stratification weights) were excluded from further analysis. Of the remaining 5993 participants, 4822 had at least one available measurement of the variables of interest (BP, cholesterol, glucose, HbA1c, weight, height). The 1171 excluded individuals were more likely to be from urban areas and in the youngest age group (i.e., 18–29 years old) and less likely to be unemployed, having a chronic disease, having children and being of Greek origin. A weighted logistic regression model adjusted for all these factors was fitted to estimate response probability.

Demographic characteristics of the study population are presented in Table 1. Percentages were estimated after weighting to resemble the structure of the adult Greek population. Overall, 51.5% were females whereas the median (IQR) age was 47.9 (34, 64) years. A total of 35.7% resided in Attica and the majority (63.8%) lived in urban areas, were married or in cohabitation (60.8%) and had kids (67.4%). About 12.9% were born in a country other than Greece. About half (46.2%) had graduated secondary or post-secondary school; household monthly income was up to 900€ for 40.1% of the population and 15.3% were unemployed.

**Table 1:** Demographic characteristics of the study population (*N* = 4822)

	N (%)	% weighted^a^
**Gender**		
Male	2065 (42.8)	48.5
Female	2757 (7.2)	51.5
**Age group (years)**		
18–29	454 (9.4)	17.7
30–39	646 (13.4)	18.3
40–49	847 (17.6)	17.7
50–59	931 (19.3)	15.6
60–69	926 (19.2)	12.7
70+	1018 (21.1)	17.9
**Residence Area**		
Attica	1123 (23.3)	35.7
Crete	292 (6.1)	5.6
Thessalonica	489 (10.1)	10.1
Thrace	289 (6.0)	3.4
Thessaly	357 (7.4)	6.7
Peloponnese	526 (10.9)	9.7
Epirus	259 (5.4)	3.2
Ionian islands	248 (5.1)	1.9
Central Greece	377 (7.8)	7.0
Macedonia	536 (11.1)	11.9
Aegean Islands	326 (6.8)	4.7
**Degree of urbanization**		
Urban	2487 (51.6)	63.8
Suburban	889 (18.4)	16.1
Rural	1446 (30.0)	20.2
**Country of Birth**		
Greece/Cyprus	4305 (89.3)	87.1
Balkans	221 (4.6)	5.9
East Europe/ Former Soviet Union	68 (1.4)	1.8
West Europe/Australia/America	88 (1.8)	1.8
Africa	32 (0.7)	0.8
Asia	35 (0.7)	1.0
Unknown	73 (1.5)	1.5
**Family status**		
Married/ Cohabitation	3226 (66.9)	60.8
Single	1535 (31.8)	37.9
Unknown	61 (1.3)	1.3
**Having kids**		
No	996 (20.7)	30.7
Yes	3741 (77.6)	67.4
Unknown	85 (1.3)	1.9
**Education**		
Primary	1774 (36.8)	28.7
Secondary/post-secondary	2055 (42.6)	46.2
Higher education	916 (19.0)	23.5
Unknown	77 (1.6)	1.6
**Household monthly Income**		
Up to 900€	1995 (41.4)	40.1
900€-1.700€	1326 (27.5)	28.5
More than 1.700€	497 (10.3)	11.7
Unknown	1004 (20.8)	19.7
**Employment status**		
Employed	1702 (35.3)	38.7
Unemployed	651 (13.5)	15.3
Retired/Household	2111 (43.8)	35.3
Other/Unknown	358 (7.4)	10.8

^a^Sampling weights with post-stratification adjustment, multiplied with the weights estimated by the inverse probability weighting method to adjust for not participating in the exams

### Main CVD risk factors under consideration

Overall, the average SBP and DPB were 129.4 mmHg and 78.1 mmHg respectively, and both were higher in men compared to women (Table 2). Fifty-four of the 4753 individuals with available measurements of BP could not be classified for hypertension status as they had normal BP, but it was unknown if they were on antihypertensive treatment. Among the remaining, the overall estimated hypertension prevalence was 39.2%, being 29.9% among those aged below 70 years. Sixty-one persons were classified as hypertensive based on taking antihypertensive drugs without having reported medical history of hypertension. Re-classifying those as non-hypertensive, the overall hypertension prevalence estimate became 38.1%. The mean total serum cholesterol level was 193.5 mg/dL with no significant differences between men and women; 59.5% had TC ≥190 mg/dL (57.1% among those aged < 70 years), 51.5% ≥ 200 and 28.5% ≥240 mg/dL. The mean fasting serum glucose was 93.1 mg/dL, being higher in men, and the mean HbA1c 5.4%, similar in men and women. Combining self-reported with fasting glucose and HbA1c measurements, the overall estimated prevalence of diabetes mellitus was 11.6%, being 7.8% among those aged < 70 years. Based only on fasting glucose criterion (available in 2384 participants) the overall diabetes prevalence was 11.5% (95% CI: 10.3–13.0) whereas based only on HbA1c criterion (available in 4343 participants) the overall diabetes prevalence was 11.1% (95% CI: 10.2–12.2). Although the prevalence was higher in men, the difference by sex did not reach the nominal statistical significance level. While there was no statistically significant difference in the mean BMI between men and women (about 28.2 kg/m^2^), the prevalence of obesity was higher in women (33.6%) compared to men (30.5%), as more men than women were in the category of overweight (Table 2). The prevalence of obesity was high even among those aged < 70 years (29.2%). The overall percentage of current smoking was high (38.2%) being even higher in men. Results after MI were similar to those reported above, with maximum difference in the estimated prevalence being always below 0.7% (data not shown). Thus, all subsequent analyses were without applying MI.

**Table 2:** Number of people with recorded data and estimated after weighting mean (95% CI) and prevalence (95% CI) of cardiovascular (CVD) risk factors overall and by sex

	Men		Women		Total	*P*-value^*^
	N	Estimates	N	Estimates	Estimates	
SBP^*^ (mm Hg) (Mean; 95% CI)	2031	131.3 (130.5, 132.2)	2722	125.2 (124.2, 126.1)	128.1 (127.5, 128.8)	< 0.001
DBP* (mm Hg) (Mean; 95% CI)	2031	80.3 (79.7, 80.9)	2722	74.9 (74.4, 75.4)	77.5 (77.1, 77.9)	< 0.001
Hypertension Prevalence (%) (Estimate; 95% CI)	2006	42.4 (39.8, 45.1)	2693	36.1 (34.0, 38.3)	39.2 (37.4, 40.9)	< 0.001
Hypertension Prevalence (% among 18–69 yrs) (Estimate; 95% C)	1573	34.8 (32.1, 37.6)	2155	25.5 (23.0, 27.0)	29.9 (28.2, 31.6)	< 0.001
HDL cholesterol (Mean; 95% CI)	1896	44.3 (43.4, 45.0)	2525	53.3 (52.5, 54.0)	48.9 (48.2, 49.5)	< 0.001
Total serum cholesterol (mg/dL) (Mean; 95% CI)	1896	192.9 (190.1, 195.6)	2525	194.2 (191.9, 196.4)	193.5 (191.6, 195.5)	0.410
Prevalence TC ≥190 mg/dL or medication (estimate; 95% CI)	1851	59.5 (56.5,62.4)	2477	60.9 (58.4,63.3)	60.2 (58.2,62.2)	0.449
Prevalence of TC ≥240 mg/dL or medication (estimate; 95% CI)	1851	27.3 (24.9,29.7)	2477	28.3 (26.4,30.3)	27.8 (26.2,29.4)	0.472
Fasting glucose (mg/dL) (Mean; 95% CI)	1065	94.8 (92.8, 96.8)	1319	91.2 (90.0, 92.5)	93.1 (91.9, 94.3)	0.002
HbA1c (%) (Mean; 95% CI)	1873	5.4 (5.4, 5.5)	2470	5.4 (5.4, 5.4)	5.4 (5.4, 5.4)	0.115
Prevalence of diabetes mellitus (%), (Estimate; 95% CI)	1888	12.4 (11.0,14.0)	2505	10.9 (9.6,12.3)	11.6 (10.7,12.7)	0.131
BMI (kg/m^2^) (Mean; 95% CI)	2039	28.3 (28.0, 28.5)	2726	28.1 (27.8, 28.4)	28.2 (28.0, 28.4)	0.395
Overweight (%) (Estimate; 95% CI)	2039	45.0 (42.5,47.5)	2726	30.6 (28.7,32.6)	37.6 (35.9,39.2)	< 0.001
Obesity (%) (Estimate; 95% CI)	2039	30.5 (28.3,32.9)	2726	33.6 (31.6,35.7)	32.1 (30.5,33.8)	0.036
Current smokers (%) (Estimate; 95% CI)	2065	44.0 (41.6,46.4)	2757	32.7 (30.5,35.0)	38.2 (36.5,39.9)	< 0.001

*Comparison between men and women

**Fig. 1:**
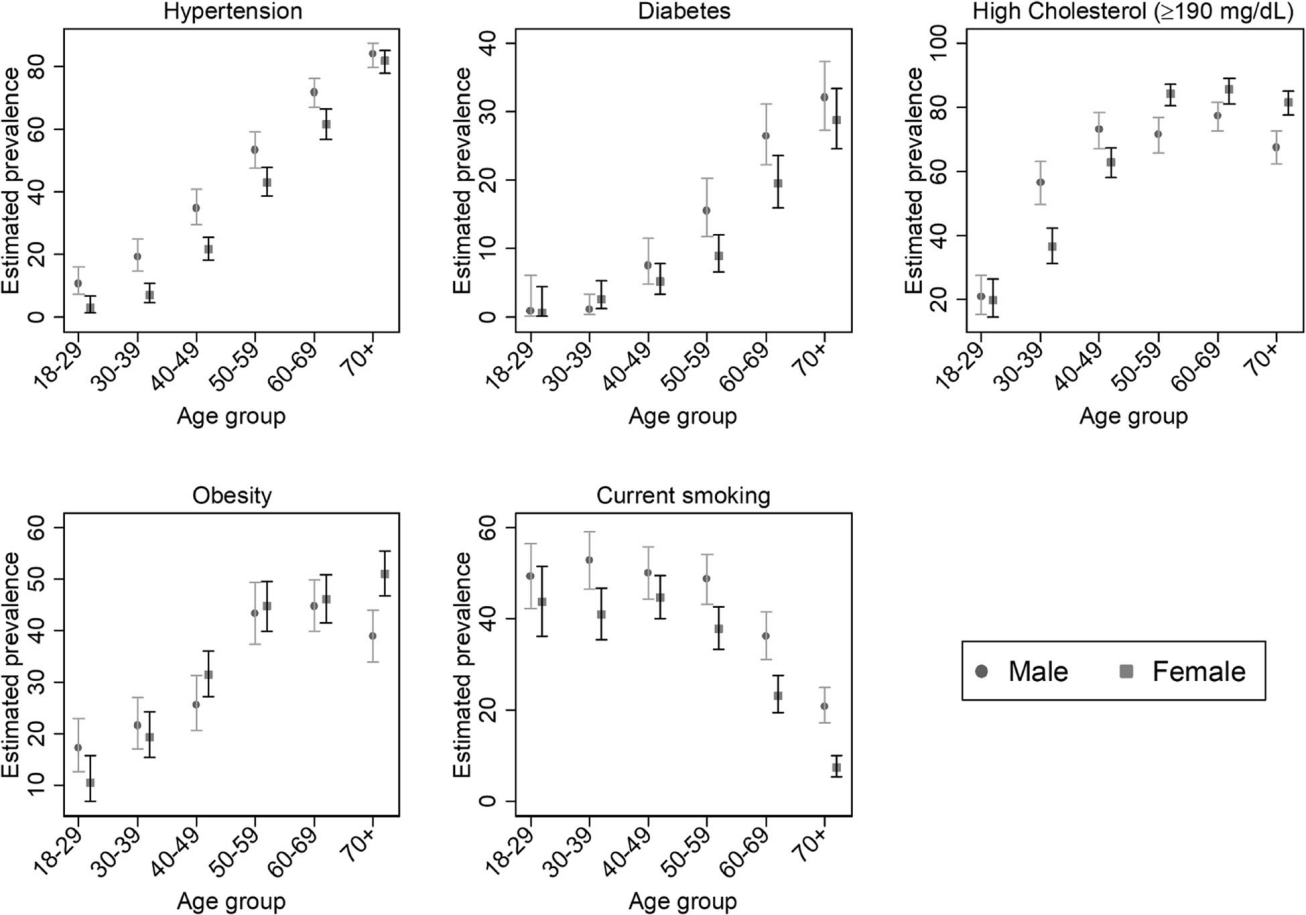
Prevalence (95% CI) of cardiovascular risk factors: hypertension, diabetes, high cholesterol, obesity, current smoking, in the general adult population, by age group and sex

Figure 1 shows the estimated prevalence of CVD risk factors under investigation by age group and sex. The prevalence of hypertension increased almost linearly with increasing age for both sexes, although the differences by sex tended to mitigate at older ages. Diabetes mellitus prevalence also increased with increasing age, similarly for both sexes. Prevalence of elevated TC levels (≥190 mg/dL) and of obesity also increased with age, although the rate of increase tended to lessen at older ages. While females had lower prevalence at younger ages, they surpassed men’s prevalence at older ages (around 50 years for elevated TC levels and around 40 years for obesity). Rates of current smoking by age followed an inverse U shape for both sexes with increasing rates up to age 40 years and decreasing rates thereafter. The difference in current smoking by sex tended to increase at older ages.

The prevalence of all risk factors (with the exception of current smoking) was higher in semi-urban and rural areas compared to urban areas. However, the differences in the prevalence by degree of urbanization became less pronounced when adjusting for age, as average age was significantly higher in semi-urban, and even more in rural areas, compared to urban ones. For diabetes, the association was even reversed, with the adjusted prevalence being higher in urban than in rural areas. The prevalence of current smoking was higher in urban areas.

Interestingly, only 14.4% of the population was not exposed to any of the investigated risk factors. About 27.5% was exposed to only one risk factor, 29.8% to 2 risk factors (in 47.0% of them smoking being one of the risk factors) whereas 28.2% were exposed to at least 3 risk factors (in 75.7% of whom hypertension and elevated cholesterol levels were involved and in 50.8% of those hypertension, elevated cholesterol levels and obesity co-existed).

## Discussion

Target 3.4 of the United Nations 3rd Sustainable Development Goal on health and well-being is to reduce premature mortality (i.e., occurring before the age of 70 years) from non-communicable diseases (CVD being the major contributor) by 33% between 2015 and 2030. To achieve this goal, good control of the modifiable risk factors at a population level is needed. This national health examination study, which is representative of the adult population in Greece, showed that the prevalence of all investigated classic CVD risk factors (hypertension, hypercholesterolemia, diabetes, obesity, smoking) were quite high among all adults (40.5, 60.2, 11.6, 32.1 and 38.2% respectively) as well as among those aged below 70 years. These results argue for urgent effective preventive measures to be taken.

Hypertension is one of the most important modifiable risk factors for CVD [15]. In our study, the prevalence of hypertension was 36.1% in women and 42.4% in men. In a recently published study, conducted in 12 high-income countries, the prevalence of hypertension ranged from 33 to 52% in women and from 39 to 59% in men [16]. It has been shown that during 2000–2010, the age-standardized prevalence of hypertension decreased by 2.6% in high-income countries and increased by 7.7% in low- and middle-income countries [17]. In Greece, in a relatively old study conducted in a small rural Greek village [9], the prevalence of hypertension was 28.5% (27% in women and 30% in men). In the Attica study, that included a representative sample of the greater Athens area, the prevalence in 2002 was 31% (25% in women and 37% in men) [8], whereas in the EPIC study [10] that included volunteers from all around the country, the age-adjusted (to the 2001 Greek population) prevalence of hypertension was 38.9% in women and 40.2% in men. Thus, overall, compared to other high-income countries, Greece is one with relatively high prevalence of hypertension; based on the limited so far data, prevalence remains at high levels with no trend towards reduction in recent years.

In the current study, about 60% of all adults living in Greece (60.9% of women and 59.5% of men) had TC levels ≥190 mg/dl or were under lipid-lowering treatment. These estimates are higher than those from previous studies in Greece. In the ATTICA study the prevalence of hypercholesterolemia (defined as TC ≥ 200 mg/dL) in 2001 was 35.2% in women and 39.9% in men [8]. These numbers should be compared to 50.7 and 51.5% found in the current study, respectively. In DEGS1, a German Health Interview and Examination Survey for adults (40–79 years old), 69% had TC levels ≥190 mg/dL (women: 72.3% and men: 65.2%) [18]. These numbers should be compared to 57.1% (56.1 and 58.1%) in the current study, respectively. Data from the National Health and Nutrition Examination Survey (NHANES: 1999–2004) [19], showed that 58.4% of non-Hispanic adult women and 59.5% of men in the USA had total blood cholesterol levels ≥200 mg/dL, estimates that are considered among the highest in the world. Although the EMENO estimates are lower than those reported from NHANES, they remain among the highest ones.

Worldwide, the prevalence of obesity has more than doubled since 1980 [20]. It is nowadays considered as apandemic whereas its association with increased risk of diabetes mellitus, hypertension and dyslipidemia is well-established, as well as its direct effect on the cardiovascular system [21]. More than one-third of adults in the US are obese (32.2%) [22], whereas this proportion is just below one-third in Europe (about 28%) [15, 21]. In Greece, a nationwide survey reported that in 2005–2006, the prevalence of self-reported obesity was 22.5% (18.2% in women and 26% in men) [23], estimates much lower than those found in the EMENO study where 33.6% of women and 30.5% of men were classified as obese. These results indicate that, from 2006 to 2014–2016, there was an alarming increase in the prevalence of obesity in Greece, particularly among women. This increase may have been driven from a change towards “modernized” diet and lifestyle. However, whether this increasing tendency is continuing or has leveled off nowadays is unknown. As socioeconomic factors also affect eating behavior and physical activity, the financial crisis in Greece may have a significant effect on public health, including obesity. Data analysis of three waves (2006, 2008 and 2011) of Hellas Health Survey, has shown that whereas the consumption of at least five portions of fruits and vegetables per day significantly decreased during the crisis among those of lower socioeconomic status, obesity prevalence (estimated based on self-reported height and weight) did not show significant trends [24]. EMENO results highlight this serious public health problem in Greece. More importantly, the prevalence of hypertension, hypercholesterolemia and diabetes, are all largely driven by the prevalence of increased BMI.

The increased prevalence of obesity worldwide is followed by increasing diabetes prevalence. According to our findings, combining fasting glucose and HbA1c criteria, the prevalence of diabetes was 11.6% with almost equal representation of both sexes (10.9% in women and 12.4% in men). The overall diabetes prevalence was of similar order using only the fasting glucose or only the HbA1c criterion (11.5 and 11.1% respectively). Unfortunately, we were not able to distinguish between diabetes type 1 and type 2. However, diabetes type 1 has been shown to be very low, around 0.24% [25]. Previous studies in Greece were based on regional or self-reported diabetes [26–28] with prevalence estimates ranging from 2.4 to 9.5%. The only nationwide study, conducted 20 years ago (1996–1999), estimating only self-reported diabetes found a prevalence of 4.3% [29]. In the Attica study (2001–2002), the diabetes prevalence was 7.9% in men and 6.0% in women [30]. Liatis et al. [25], using the electronic prescription database of the National Organization for Health Care Services Provision estimated a prevalence of medication-prescribed diabetes of 8.2% in adults, an estimate compatible with ours given that in our study both diagnosed and undiagnosed adults irrespectively of their treatment were included. Diabetes prevalence in Greece is similar to that in Spain (10.6%) [21], lower than that in China (16.8%) and the US (19.3%) [31], but relatively higher compared to that in other European countries like Germany, where diabetes prevalence was found equal to 9.2% [32]. The increased prevalence of obesity as well as the financial crisis may have contributed to this increased diabetes prevalence in Greece. Although diabetes prevalence was substantially increased in the elderly (about 30%) it was high even among those aged below 70 years (7.8%). However, comparison of our results with those previously reported should be interpreted cautionary, as different definitions are used in different studies.

According to the European Network for Smoking Prevention [33], Greece is among the countries with the largest consumption of cigarettes per capita in Europe and the highest percentages of female smokers. Based on EMENO findings, current smoking prevalence during 2014–2016 was 37.8% overall, being 44% in men and 32.4% in women. In ATTICA study (2001–2002), 39% of women and 51% of men were current smokers [8]. Fillipidis et al. [24] found that smoking prevalence decreased from 42.6 to 38.1% duringthe financial crisis 2008–11. Combining these results with ours, we can conclude that, the tendency for decreasing smoking prevalence has leveled off, remaining constant after 2011, as our estimate is almost identical to that reported for 2011, probably reflecting the failure of anti-smoking legislation. Current smoking prevalence is much higher than the average in other European countries (28%) [33] and in US (24.4% among non-Hispanic white [19]). Recently, new legislative measures to ban smoking in public and work-places were taken. Although they were not combined with measures aiming to help people quit smoking, it is expected that they will lead to substantial prevalence reduction.

Our study is also subject to some limitations. The sample was restricted to those who had available measurements for the investigated risk factors. The participants differed from those excluded in several characteristics. To adjust for that, we applied the inverse probability weighting method limiting the potential of induced bias. However, the possibility of unmeasured confounders cannot be excluded. A small proportion of the included individuals had missing values in one or more of the investigated risk factors. Sensitivity analysis after imputing missing values though, gave the same results as the main analysis. Institutionalized individuals, homeless and migrants living in detention camps were excluded from the reference population which may have slightly underestimated the study findings. As various classes of BP medications may be prescribed for other than hypertension conditions, classifying people as hypertensive based only on taking antihypertensive drugs ignoring they had not a self-reported medical history of high BP, may lead to overestimate prevalence rate. However, in a sensitivity analysis, reclassifying those participants as non-hypertensive, changed the overall prevalence estimate from 39.2 to 38.1%, so even if there was an upwards bias, this should not exceed 1–1.5%.

## Conclusions

This is the first nationwide study in Greece, which included a representative sample of the non-institutionalized adult population and reported the prevalence of modifiable CVD risk factors. Our results showed that all modifiable CVD risk factors investigated in this study (hypertension, hypercholesterolemia, diabetes, obesity, smoking) are very common and without signs of future decline, apart maybe of smoking. What is more worrying, quite often these risk factors co-existed with 28.2% of the population been exposed to at least 3 risk factors. It has been shown that in recent years the decline in CVD mortality has been slowed down in most high-income countries including Greece [34], which, however, never reached the previous high rates of decline observed in other high-income countries. In Greece, control of the key CVD risk factors is undoubtfully suboptimal. There is thus, a high preventive potential. Actions aiming at CVD risk factor prevention, detection and control should be urgently designed and applied at both health care system and societal level. Our estimates could contribute in updating CVD risk score estimates overall and by sex and age group in the Greek population.

## Data Availability

The data that support the findings of this study could be shared after interested researchers submit a concept form to the chair of the EMENO steering committee (Giota Touloumi, email: gtouloum@med.uoa.gr) after the concept form been approved by the EMENO steering committee.

## Abbreviations

EMENO: National Survey of Morbidity and Risk Factors
BP: Blood Pressure
TC: Total Cholesterol
BMI: Body Mass Index
HbA1c: Glycate Hemoglobin
CVD: Cardiovascular Diseases
SBP: Systolic Blood Pressure
DBP: Diastolic Blood Pressure
MI: Multiple Imputation
